# Rapid and sustained cognitive recovery in APP/PS1 transgenic mice by co-administration of EPPS and donepezil

**DOI:** 10.1038/srep34165

**Published:** 2016-10-31

**Authors:** Hye Yun Kim, Hyunjin Vincent Kim, Dongkeun K. Lee, Seung-Hoon Yang, YoungSoo Kim

**Affiliations:** 1 Convergence Research Center for Dementia and Center for Neuro-Medicine, Brain Science Institute, Korea Institute of Science and Technology, Hwarangno 14-gil 5, Seongbuk-gu, Seoul, Republic of Korea; 2 Biological Chemistry Program, Korea University of Science and Technology, 217 Gajungro, Yuseong-gu, Daejeon, Republic of Korea; 3 Department of Pharmaceutical Science, College of Pharmacy, Kyung Hee University, 26 Kyungheedae-ro, Dongdaemun-gu, Seoul, Republic of Korea

## Abstract

Alzheimer’s disease (AD) is a neurodegenerative disease characterized by sequential progression of pathological events, such as aggregation of amyloid-β proteins, followed by outward symptoms of cognitive impairments. Given that a combination of different therapeutic strategies often provides more rapid and effective outcomes in diverse areas of clinical treatment, we hypothesized that administration of anti-amyloid drugs with cognitive enhancers would result in synergistic effects in AD treatment. Here, we co-administered 4-(2-hydroxyethyl)-1-piperazinepropane-sulphonic acid (EPPS), an amyloid-clearing chemical, and donepezil, an acetylcholinesterase inhibitor, to determine whether they could serve complementary roles for each other in regards to AD treatment. We found that oral administration of these two molecules led to a rapid and consistent cognitive improvement in APP/PS1 transgenic mice. Although there was no evidence for synergistic effects, our results indicated that EPPS and donepezil function complementary to each other without altering their individual effects. Thus, the combined use of disease-modifying and symptomatic relief drugs may be a promising approach in the treatment of AD.

At present, the most common therapeutic options clinically available for Alzheimer’s disease (AD) are acetylcholinesterase inhibitors to provide symptomatic reliefs^1^,^2^,^3^. Despite benefits of these symptomatic drugs, there is an unmet need to halt the fatal neurodegeneration of AD because neurotoxic pathological markers, such as amyloid-β (Aβ) and tau aggregates, still damage the Alzheimer brain. Many studies have focused on fidning drug candidates that can alter the pathogenesis of AD in the last three decades^4^,^5^. Nonetheless, brain atrophy associated with cognitive deficits could not be recovered by modulating pathological culprits^6^. Thus, both disease-modifying and symptomatic relief drugs are necessary as an intervention to effectively treat AD. We hypothesized that a combination of anti-amyloid and anti-acetylcholinesterase therapeutic strategies would complement each other and result in a relatively prompt symptom improvement along with Aβ clearance taking place in the Alzheimer brain with a sustained symptomatic control owing to the action of the disease-modifying drug. Herein, we administered 4-(2-hydroxyethyl)-1-piperazinepropanesulphonic acid (EPPS), for its disease-modifying effect, and donepezil, for its symptomatic relief, together to aged APPswe/PS1-dE9 (amyloid precursor protein/presenilin protein 1) mice (APP/PS1). This mouse model produces elevated levels of human Aβ by expressing mutant human APP and PS1, which leads to development of Aβ plaques and AD-like cognitive impairments from 6 months of age^7^,^8^. EPPS was previously reported to directly disaggregate Aβ oligomers and plaques back into inert monomers in the brains of APP/PS1 mice^7^. Donepezil directly inhibits acetylcholinesterase in the cholinergic synapse to increase acetylcholine concentration in the brain, thereby producing rapid symptomatic relief^9^. During the study, we performed behaviour tests to include Y-maze and fear-conditioning tasks to assess the cognitive recovery of the mice. We also measured the levels of Aβ plaques and oligomers by histochemistry and sandwich enzyme-linked immunosorbent assay (ELISA).

## Result

### EPPS restores cognitive function of APP/PS1 mice within 4 weeks

**Figure 1:**
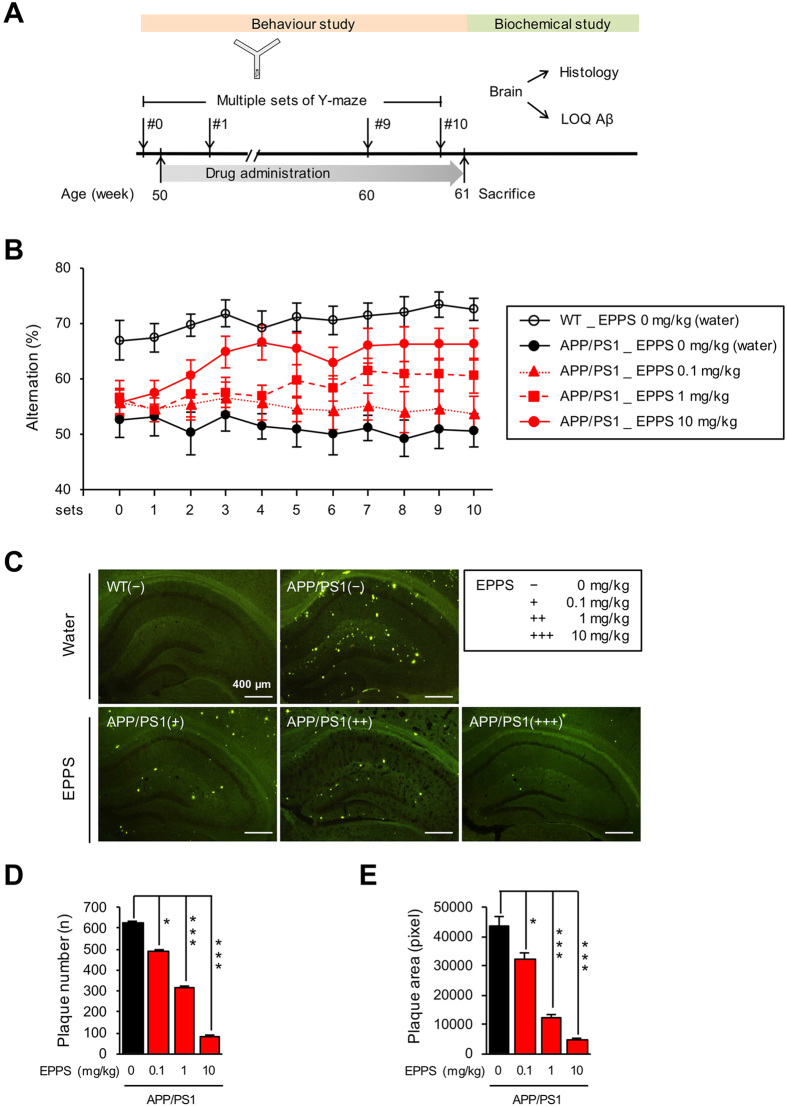
Administration of EPPS in APP/PS1 double transgenic mice. EPPS in drinking water was administered to 50-week-old APP/PS1 male mice (water, n = 5; 0.1, 1, and 10 mg/kg/day of EPPS, n = 7, 9, 9) for 10 weeks and compared to age-matched wild type (WT, n = 11). Y-maze tests were performed at every week for 10 weeks. After the last set of Y-maze test, Aβ plaques in brains were stained by thoflavin-S. (**A**) Experiment schedule (LOQ: level of quantification). (**B**) (%) Alternations of Y-maze tests. Multiple sets of Y-maze tests were analysed by two-way repeated measures ANOVA between wild type and APP/PS1 mice with various EPPS dosages followed by Bonferroni’s *post-hoc* test (See in Supplementary Table 1 for statistical analyses). (**C**) Hippocampal region (bregma −1.58~−2.18 mm, 6 slides/mouse) of the mouse brain with thoflavin-S staining (scale bar, 400 μm). (**D**) Quantifications of number and (**E**) area of stained Aβ plaques. ImageJ software was used to quantify numbers and pixel areas of Aβ plaques. Numbers of mice for biochemical analyses are described in Methods. One-way ANOVA with Bonferroni’s *post-hoc* comparisons were performed in statistical analyses. All the error bars represent the SEMs. (*P < 0.05, **P < 0.01, ***P < 0.001).

Aged APP/PS1 model mice (male, 50 weeks of age) and their age-matching wild-type (WT, n = 11) controls were used in this study. The APP/PS1 model is known to show elevated levels of human Aβ by 6–7 months and impaired memory after 8 months of age. In the previous study, we observed clearance of Aβ aggregates and recovery of cognitive impairments in the same mouse model by long-term administration of EPPS in 10, 30 and 100 mg/kg/day^7^. To determine the minimum duration and dosage of EPPS administration for its therapeutic effect, the lower dosages of EPPS (0, 0.1, 1, and 10 mg/kg/day, n = 5, 7, 9, and 9, respectively) were administered orally to APP/PS1 mice daily for 10 weeks. We subjected the mice to weekly Y-maze tests during the EPPS treatment and recorded the sequences of arm entries to analyse the percent alternations reflecting spatial working memory of mice (Fig. 1A)^10^. Two-way repeated measures ANOVA test showed a significant genotype effect (*F*(1, 14) = 72.86, *P* < 0.0001) and EPPS effect (*F*(3, 26) = 21.88, *P* < 0.0001). From APP/PS1 mice treated with EPPS of 1 and 10 mg/kg, the percent alternations were shown to be significantly different from those of non-treated APP/PS1 mice by week 4 in a dose-dependent manner, suggesting recovery in their short-term working memory (Supplementary Table 1). APP/PS1 mice administered with 0.1 mg/kg of EPPS, however, did not show behavioural improvements. The level of percent alternations stayed elevated until the last set of Y-maze tests conducted on week 10 (Fig. 1B).

To assess the Aβ clearance by EPPS in the brain, mice were sacrificed after the completion of Y-maze tests and brains were extracted for histochemical analyses. The Aβ plaques in the hippocampus of brain slices (6 slides per mouse) were visualised using thioflavin-S staining (bregma −1.58~−2.18 mm)^7^. Compared to the non-treated APP/PS1 mice, the amount of Aβ plaques was dramatically reduced in the hippocampal region of APP/PS1 mice treated with 0.1, 1, and 10 mg/kg of EPPS (Fig. 1C). Quantitative analysis indicated that number and pixel area of Aβ plaques were also decreased in a dose-dependent manner, with EPPS concentration of 0.1, 1 and 10 mg/kg showing significant reductions compared to non-treated APP/PS1 mice (number: *P* = 0.0298, *P* < 0.0001, and *P* < 0.0001; area: *P* = 0.0253, *P* < 0.0001, and *P* < 0.0001).

Collectively, EPPS in 1 and 10 mg/kg/day significantly recovered the cognitive ability of APP/PS1 mice up to the level of WT mice with reduced amount of Aβ plaques in the brain and sustained the effects since the 4 weeks of oral administration.

### Donepezil rapidly enhances but does not sustain the cognitive function of APP/PS1 mice

**Figure 2:**
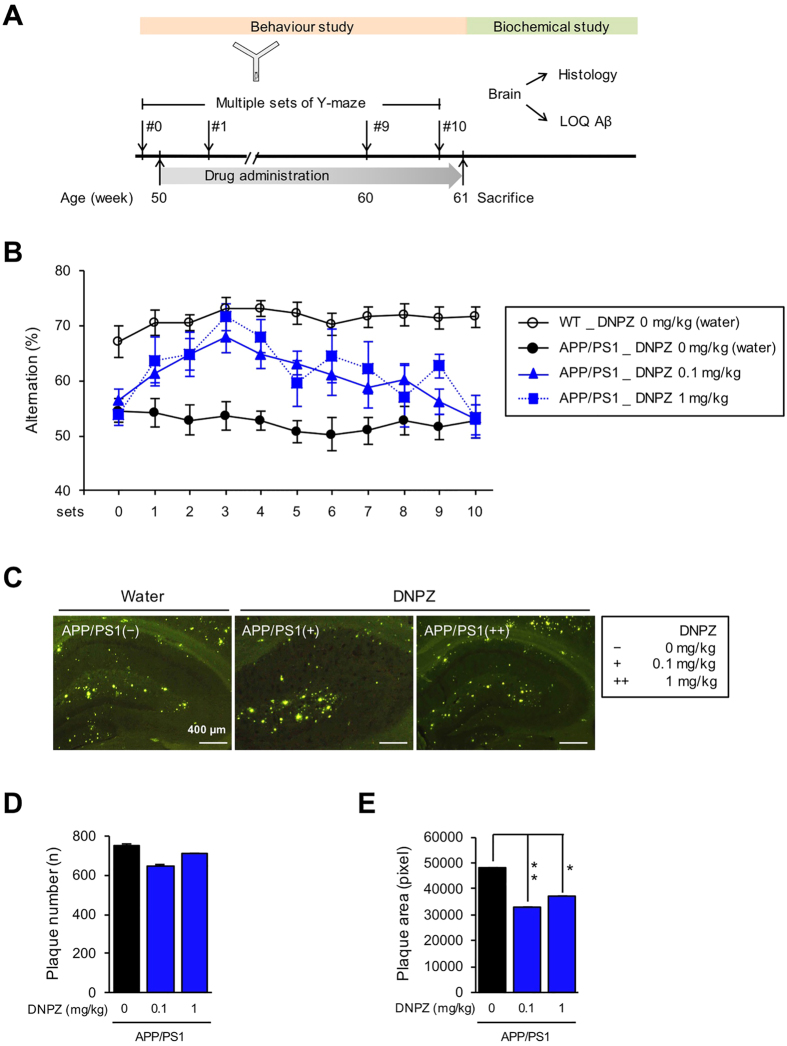
Administration of donepezil in APP/PS1 double transgenic mice. Donepezil (DNPZ) in drinking water was administered to 50-week-old APP/PS1 male mice (water, n = 9; 0.1 and 1 mg/kg/day of donepezil, n = 6 and 5) for 10 weeks and compared to age-matched wild type (WT, n = 13). Y-maze tests were performed at every week for 10 weeks. After the last set of Y-maze test, Aβ plaques in brains were stained by thoflavin-S. (**A**) Experiment schedule (LOQ: level of quantification). (**B**) (%) Alternations of Y-maze tests. Multiple sets of Y-maze tests were analysed by two-way repeated measures ANOVA between wild type and APP/PS1 mice with various donepezil dosages followed by Bonferroni’s *post-hoc* test (See in Supplementary Table 2 for statistical analyses). (**C**) Hippocampal region (bregma −1.58~−2.18 mm, 6 slides/mouse) of the mouse brain with thoflavin-S staining (scale bar, 400 μm). (**D**) Quantifications of number and (**E**) area of stained Aβ plaques. ImageJ software was used to quantify numbers and pixel areas of Aβ plaques. Numbers of mice for biochemical analyses are described in Methods. One-way ANOVA with Bonferroni’s *post-hoc* comparisons were performed in statistical analyses. All the error bars represent the SEMs. (*P < 0.05, **P < 0.01, ***P < 0.001).

To observe changes in the therapeutic efficacy of donepezil over the course of the drug administration, aged APP/PS1 model mice (male, 50 weeks of age) and their age-matching wild-type (WT, n = 13) controls were used. Donepezil (0, 0.1, and 1 mg/kg/day, n = 9, 6, and 5, respectively)^11^ was administered to APP/PS1 mice groups for 10 weeks and Y-maze test was performed (Fig. 2A). Two-way repeated measures ANOVA test showed a significant genotype effect (*F*(1, 20) = 234.10, *P* < 0.0001) and donepezil effect (*F*(2, 17) = 22.49, *P* < 0.0001). As a result, percent alternations of donepezil-treated APP/PS1 mice were promptly increased starting on week 1, suggesting the rapid effect of donepezil in the improvement of spatial working memory. From week 2, APP/PS1 mice treated with 0.1 and 1 mg/kg of donepezil showed percent alternations significantly different from non-treated APP/PS1 mice (*P* = 0.0051 and *P* = 0.0186, respectively), and that of APP/PS1 mice treated with 1 mg/kg reached the level similar to that of WT mice by week 3 (Supplementary Table 2).

At week 4 and 5, however, efficacy of donepezil started to significantly diminish for both 0.1 and 1 mg/kg compared to WT mice (all *P* > 0.05). The efficacy continued to decrease in donepezil-treated mice, resulting in their percent alternations declining back to the level of non-treated, cognitively impaired APP/PS1 mice group by week 11 (Fig. 2B).

Previous study inferred that donepezil has anti-amyloidogenic effects while serving its intended role as a symptomatic drug to alleviate cognitive deficits^11^. To determine whether donepezil alters the amount of Aβ plaques, mice were sacrificed after the last set of Y-maze tests and brains were extracted and sliced for thioflavin-S staining (6 slides per mouse). Brain images showed decreased amount of Aβ plaques when 1 mg/kg of donepezil was administered to APP/PS1 mice (Fig. 2C). Quantification analyses showed that area but not number of Aβ plaques were decreased (*P* = 0.0072, and *P* = 0.0115, respectively) compared to non-treated APP/PS1 mice, suggesting that donepezil exhibits weak inhibitory effect against Aβ aggregation (Fig. 2D,E). Collectively, donepezil administration resulted in rapid recovery of cognitive ability within a week, which started to decline gradually over the course of the drug administration.

### Co-administration of EPPS and donepezil results in rapid and sustained recovery of cognitive function

**Figure 3:**
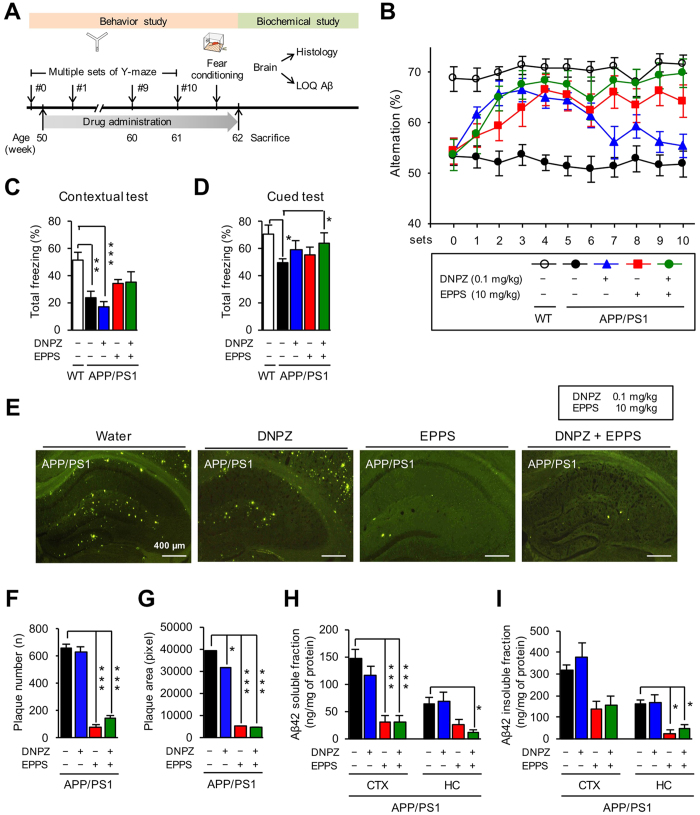
Co-administration of EPPS and donepezil in APP/PS1 double transgenic mice. EPPS and donepezil (DNPZ) in drinking water were administered to 50-week-old APP/PS1 male mice (water, n = 12; 10 mg/kg/day of EPPS, n = 8; 0.1 mg/kg/day of donepezil, n = 10; and EPPS/donepezil co-administration, n = 9) for 10 weeks and compared to age-matched wild type (WT, n = 19). Y-maze tests were performed at every week for 10 weeks. Then, fear-conditioning tests were performed on week 11, and Aβ plaques and oligomers in brains were stained and quantified by thoflavin-S and ELISA, respectively. (**A**) Experiment schedule (LOQ: level of quantification). (**B**) (%) Alternations of Y-maze tests. Multiple sets of Y-maze tests were analysed by two-way repeated measures ANOVA between wild type and APP/PS1 mice with various mixtures of EPPS and donepezil dosages followed by Bonferroni’s *post-hoc* test (See in Supplementary Table 3 for statistical analyses). (**C**) Contextual test and (**D**) cued test of fear-conditioning. (**E**) Hippocampal region (bregma −1.58~−2.18 mm, 6 slides/mouse) of the mouse brain with thoflavin-S staining (scale bar, 400 μm). (**F**) Quantifications of number and (**G**) area of stained Aβ plaques. ImageJ software was used to quantify numbers and pixel areas of Aβ plaques. (**H**) Quantifications of soluble Aβ42 and (**I**) insoluble Aβ42 by ELISA analyses (CTX: cortex, HC: hippocampus). Numbers of mice for biochemical analyses are described in Methods. One-way ANOVA with Bonferroni’s *post-hoc* comparisons were performed in statistical analyses. All the error bars represent the SEMs. (*P < 0.05, **P < 0.01, ***P < 0.001).

To observe whether EPPS and donepezil are complementary to each other in the AD treatment, we used aged APP/PS1 model mice (male, 50 weeks of age) and their age-matching wild-type (WT, n = 17) controls. APP/PS1 mice were administered with 10 mg/kg of EPPS and 0.1 mg/kg of donepezil – concentrations of which were determined to show substantial effects of drugs based on aforementioned behaviour tests – by drinking water for 11 weeks (n = 9). We performed Y-maze tests weekly from week 0 to 10 during the drug administration (Fig. 3A). Two-way repeated measures ANOVA test showed a significant genotype effect (*F*(1, 27) = 366.80, *P* < 0.0001) and drug effect (*F*(3, 35) = 40.99, *P* < 0.0001). Compared to the non-treated APP/PS1 mice (n = 12), percent alternations were dramatically increased in EPPS/donepezil co-administered APP/PS1 mice that the level of working memory reached up to that of WT mice by week 2 (*P* = 0.0157), which were more rapid changes than those of APP/PS1 mice treated only with EPPS (10 mg/kg, n = 8) (Supplementary Table 3). EPPS/donepezil co-administered mice also showed elevated level of percent alternations until the last set of Y-maze test on week 10, whereas APP/PS1 mice treated only with donepezil (0.1 mg/kg, n = 10) showed cognitive enhancement starting to decline on week 4. Noticeably, the level of cognitive recovery in EPPS/donepezil co-administered APP/PS1 mice was numerically higher than those treated only with EPPS throughout 10 weeks of Y-maze tests, yet we did not find statistically significant differences (Fig. 3B).

After the completion of Y-maze tests, we performed fear-conditioning tests on week 11 to assess the emotion-associated learning ability of mice in a longer time period^10^. We subjected mice to the contextual test to measure freezing responses related to both hippocampus and amygdala, and performed the cued test to measure freezing responses related to amygdala. In contextual tests (Fig. 3C), donepezil administration in APP/PS1 mice had no effect in total freezing responses compared to WT mice (*P* = 0.0005), suggesting that donepezil does not affect the hippocampal-dependent contextual memory function. These results from contextual tests performed on week 11 are consistent with the declined effect of donepezil we observed in the last set of the Y-maze test. For EPPS-administered APP/PS1 mice, either singly or with donepezil, there were increasing trends of total freezing responses, but they were not statistically significant. In cued tests (Fig. 3D), APP/PS1 mice administered with either donepezil or EPPS showed trends of increases in total freezing responses, yet they were not statistically significant compared to WT mice. However, APP/PS1 mice co-administered with EPPS and donepezil showed significantly increased total freezing responses compared to non-treated APP/PS1 mice (*P* = 0.0430), suggesting the co-administration improved the amygdala-related memory function of APP/PS1 mice.

After the behavioural tests were completed, mice were sacrificed and their brains were extracted to measure the amount of Aβ plaques and oligomers in the brain. Brain sections were stained with thioflavin-S to visualise Aβ plaques. Compared with non-treated APP/PS1 mice, those treated with either EPPS alone or EPPS with donepezil showed less Aβ plaques (Fig. 3E). Quantifications of Aβ plaques observed in brain images indicated that number and pixel area of Aβ plaques were significantly lower in these two groups (*P* < 0.0001 for all cases) than in non-treated APP/PS1 mice (Fig. 3F,G). We further performed sandwich ELISA using the brain tissues to observe the amount of soluble and insoluble Aβ42. Hippocampal and cortical lysates were prepared with RIPA lysis (soluble fraction) and guanidine (insoluble fraction) buffers. As results, the amounts of Aβ42 in both soluble and insoluble fractions were shown to be lower in the mice administered with EPPS alone or EPPS with donepezil (Fig. 3H,I).

Collectively, our results indicated that co-administration of EPPS and donepezil in APP/PS1 mice recovered the cognitive and learning abilities more rapidly and consistently compared to when each molecule was singly administered, and amount of Aβ plaques and oligomers were decreased as well. However, reduction of Aβ plaques and oligomers even when EPPS was singly administered suggested that donepezil does not increase the anti-amyloid effect of EPPS.

## Discussion

AD is characterized by severe symptoms of gradual cognitive impairments. Clinically available for AD patients are symptomatic drugs, such as acetylcholinesterase inhibitors, for dramatic alleviation of the outward symptoms^2^,^3^. Yet, given that underlying causes of the disease precede the onset of symptoms, disease-modifying drugs targeting pathological biomarkers, including Aβ plaques and oligomers, would provide further benefits for AD patients^12^.

In this study, we examined whether disease-modifying and symptomatic drugs could complement each other when co-administrated in transgenic AD mouse model. EPPS was used to clear out Aβ aggrega tes from the brain as it was previously found to be safe and tolerable when orally administered to APP/PS1 mice^7^. To alleviate cognitive deficits, donepezil was selected as it has been wildly used in clinics for long period of time and reported with minimal adverse drug reactions^9^,^13^.

Our results showed that effect of EPPS on cognitive recovery was maintained at an elevated state throughout the period of administration, yet it took several weeks to observe significant effect of the molecule. Given that the previous study of EPPS showed that the AD-like behaviors are highly correlated with the level of Aβ aggregates in the brain^7^, slow effect of the EPPS we observed from APP/PS1 mice could be attributed to the time required to dissemble Aβ aggregates. On the other hand, donepezil administration led to more prompt behavioural changes that APP/PS1 mice showed cognitive recovery; however, its effect was transient, possibly due to the progression of the disease even with the use of the symptomatic drug. Co-administration of EPPS and donepezil was aimed at simultaneously targeting pathological and symptomatic reliefs, and it resulted in rapid and sustained cognitive recovery, suggesting that two molecules of different modes of actions complemented each other.

Drug-drug interactions must be considered during the development of cocktail therapy. Despite the absence of synergic effects between EPPS and donepezil, their co-administration did not reduce or alter each other’s therapeutic effects on cognition. Additional studies are warranted to determine whether co-administration of EPPS and donepezil could deliver therapeutic effects to AD patients.

## Methods

### Reagents

4-(2-hydroxyethyl)-1-piperazinepropanesulfonic acid (EPPS), donepezil hydrochloride monohydrate, thioflavin-S, and protease inhibitor cocktail were from Sigma-Aldrich (St. Louis, MO). Deionized water was generated by a Milli-Q plus from Millipore (Bedford, MA). A RIPA buffer and phosphate buffered saline (PBS buffer) were purchased from ThermoFisher. (Roxkford, IL). Immunoassay kits for insoluble fraction of Aβ quantifications were used from Invitrogen (KHB3442, Camarillo, CA).

### AD transgenic mice preparation

Doubly mutated transgenic mice (male), originally obtained from Jackson Laboratory (USA; strain name: B6C3-Tg (APPswe/PS1-dE9) 85Dbo/J; stock number 004462), were supplied from Hanmi Pharm Co., Ltd. (Gyeonggi, Korea) and Medifron DBT (Gyeonggi, Korea). These APP/PS1 transgenic mice were maintained as double hemizygotes by crossing with wild type mice on a C57BL/6J × C3H backgrounds train. All mice were housed 4 to 5 per plastic cage in a room maintained at 21 ± 1 °C, with an alternating 12 hr light-dark cycle and free access to food and water. Behavioural experiments were carried out in a sound-attenuated and air-regulated experiment room, following at least 30 min of habituation time. All of the animal experiments were carried out in accordance with the National Institutes of Health guide for the care and use of laboratory animals (NIH Publications No. 8023, revised 1978). The animal studies were approved by the Institutional Animal Care and Use Committee of Korea Institute of Science and Technology.

### EPPS and donepezil administration

EPPS and donepezil in drinking water were freely administered for about 3 months (11–12 weeks) with different dosage (0.1, 1, or 10 mg/kg) to 12-month-old (50 weeks of age) wild type and APP/PS1 transgenic male mice exhibiting severe AD-like behaviours and Aβ plaque deposits in brains. For the exact administration of EPPS and donepezil, their dosages were calculated based on the average daily water consumption recorded in each cage, and they were confirmed by re-calculations once a week. Fresh drinking water was provided daily. The experimental schedule is shown in Figs 1, 2 and 3. Over the course of the oral administration, we performed Y-maze tests every week to examine the efficacy of drugs and performed fear-conditioning tests after the last set of Y-maze. After all behaviour tests, mice were sacrificed for further biochemical analyses. Numbers of mice used in the experiments of EPPS administration (Fig. 1) were: 11 (WT, water), 5 (APP/PS1, water), 7 (APP/PS1, 0.1 mg/kg/day), 9 (APP/PS1, 1 mg/kg/day), and 9 (APP/PS1, 10 mg/kg/day). Numbers of mice used in the experiments of donepezil administration (Fig. 2) were: 13 (WT, water), 9 (APP/PS1, water), 6 (APP/PS1, 0.1 mg/kg/day), and 5 (APP/PS1, 1 mg/kg/day). Numbers of mice used in the experiments of EPPS/donepezil co-administration (Fig. 3) were: 17 (WT, water), 12 (APP/PS1, water), 8 (APP/PS1, 10 mg/kg/day of EPPS), 10 (APP/PS1, 0.1 mg/kg/day of donepezil), and 9 (APP/PS1, 10/0.1 mg/kg/day of EPPS/donepezil).

### Multiple trials of Y-maze test

Y-maze test is used to assess cognitive changes and short-term spatial working memory (by spontaneous alternation) and exploratory activity (by total number of arm choices) of mice placed into a black Y-maze^10^. The Y-maze is a three-arm horizontal maze (40 cm-long and 10 cm-wide with 12 cm-high walls) in which the arms are symmetrically disposed at 120° angles from one another. Mice were placed at the end of one arm and allowed to move freely through the maze during an 8-min session. The number of total arm choices and sequence of arm choices were recorded. The percent alternation is defined by proportion of arm choices that differ from the last 2 choices. Before each trial, the interior of the maze was sprayed with a 70% ethanol solution to erase any scent cues. To evaluate the efficacy of drugs in a time-dependent manner, Y-maze tests were performed every week during the drug administration periods^14^.

### Fear conditioning test

Fear-conditioning test measures the ability of mice to learn and remember an association between aversive experience and environmental cue^10^,^15^. The hippocampal lesions interfere with contextual conditioning but not the cued, whereas amygdala lesions interfere with both contextual and cued conditionings^16^. Both contextual and cued tasks were carried out 4 days after the last set of Y-maze test. On the first day, mice were placed into a cued box for 5 min to habituate and explore. Next day, for training (fear-conditioning phase), mice were placed into a sound-attenuating standard operant chamber (Coulbourn, USA). After 3 min of adjustment in the chamber, 30-sec of tone (3 kHz, 85 dB) was delivered with a foot shock (0.5 mA) at the last 1 sec of tone stimulus. This training was repeated two times continuously with 90-sec intervals. After 24 hr, mice were subjected to the same chamber for contextual retention test. Its movements were recorded using a video camera for 5 min to score freezing responses, which were defined as lack of movements except for respiration. Cued fear-conditioning was performed on the final day of fear conditioning study. After placed into the cued box, mice were allowed to explore for 3 min and the conditioning tone was delivered for 1 min. The responses were recorded using a video camera to score freezing.

### Thioflavin-S staining

After the behaviour tests, mice were deeply anesthetized with a blend of tiletamine. HCl, zolazepam. HCl (80 mg/kg, IP, Zoletil 50®, Virbac, France), and xylazine (20 mg/kg, IP, Rompun®, Bayer Pharma, Germany) and perfused with 0.9% saline followed by ice-cold 4% paraformaldehyde. Excised brains were post-fixed overnight in 4% paraformaldehyde at 4 °C and immersed in 30% sucrose for 48 hr for cryoprotection. Coronal sections (6 slides/animal) of the brain (30 μm) were cut with a Cryostat (Microm HM 525, Thermo Scientific, Walthman, MA, USA) and stained with thioflavin-S, which wasprepared at 500 μM in 50% of ethanol, for 7 min to visualise Aβ plaques. Then, brain sections were soaked sequentially into 100, 95 and 90% of ethanol for 10 sec and then moved into PBS to remove a non-specific binding of the dye. The images of hippocampal region along the AP axis (bregma −1.58~−2.18 mm) were taken on an Olympus fluorescent microscope using a GFP filter set^17^. ImageJ software was used to quantify numbers and pixel areas of Aβ plaques. Numbers of mice used in the brain staining of EPPS administration (Fig. 1) were: 5 of 11 (WT, water), 2 of 5 (APP/PS1, water), 3 of 7 (APP/PS1, 0.1 mg/kg/day), 4 of 9 (APP/PS1, 1 mg/kg/day), and 4 of 9 (APP/PS1, 10 mg/kg/day). Numbers of mice used in the brain staining of donepezil administration (Fig. 2) were: 6 of 13 (WT, water), 4 of 9 (APP/PS1, water), 3 of 6 (APP/PS1, 0.1 mg/kg/day), and 2 of 5 (APP/PS1, 1 mg/kg/day). Numbers of mice used in the brain staining of EPPS/donepezil co-administration (Fig. 3) were: 8 of 17 (WT, water), 6 of 12 (APP/PS1, water), 4 of 8 (APP/PS1, 10 mg/kg/day of EPPS), 5 of 10 (APP/PS1, 0.1 mg/kg/day of donepezil), and 4 of 9 (APP/PS1, 10/0.1 mg/kg/day of EPPS/donepezil).

### Quantifications of the Aβ levels

Levels of soluble and insoluble Aβ were quantified following the procedures of Kawarabayashi *et al*.^18^. Hippocampal and cortical regions of the brain were dissected separately and homogenized in ice-cold lysis buffer, RIPA, containing 1x protease inhibitors cocktail. Homogenized tissues were incubated in ice for 20 min and centrifuged at 14,000 rpm, 4 °C for 30 min. The supernatant (soluble fraction) of brain lysates was analyzed with Aβ42 ELISA kit purchased from Invitrogen (KHB3442). To obtain Aβ insoluble fraction in brain lysates, a guanidine buffer (5 mM guanidine-HCl, 50 mM Tris-HCl, pH 8.0) containing 1x protease inhibitors cocktail was added to the pellet of brains lysates and incubated at room temperature for 3 hr with shaking. Then, the mixture was centrifuged at 4 °C for 2 hr to obtain Aβ insoluble fraction in supernatant. Levels of insoluble fractions were measured using the Aβ42 ELISA kit. Protein concentrations of soluble and insoluble fractions were determined by Bradford protein assay. Based on the results, 20 μg of soluble and 10 μg of insoluble fractions were used for ELISA analyses. Numbers of mice used in the brain lysates of EPPS administration (Fig. 1) were: 6 of 11 (WT, water), 3 of 5 (APP/PS1, water), 4 of 7 (APP/PS1, 0.1 mg/kg/day), 5 of 9 (APP/PS1, 1 mg/kg/day), and 5 of 9 (APP/PS1, 10 mg/kg/day). Numbers of mice used in the brain lysates of donepezil administration (Fig. 2) were: 7 of 13 (WT, water), 5 of 9 (APP/PS1, water), 3 of 6 (APP/PS1, 0.1 mg/kg/day), and 3 of 5 (APP/PS1, 1 mg/kg/day). Numbers of mice used in the brain lysates of EPPS/donepezil co-administration (Fig. 3) were: 9 of 17 (WT, water), 6 of 12 (APP/PS1, water), 4 of 8 (APP/PS1, 10 mg/kg/day of EPPS), 5 of 10 (APP/PS1, 0.1 mg/kg/day of donepezil), and 5 of 9 (APP/PS1, 10/0.1 mg/kg/day of EPPS/donepezil).

### Statistical analysis

Graphs were obtained with GraphPad Prism 6. Data from multiple sets of Y-maze tests were analysed by two-way repeated measures ANOVA with Bonferroni’s *post-hoc* comparisons. Data from other tests were analysed by one-way ANOVA with Bonferroni’s *post-hoc* comparisons (*P < 0.05, **P < 0.01, ***P < 0.001). The error bars represent the SEMs.

## Additional Information

**How to cite this article**: Kim, H. Y. *et al*. Rapid and sustained cognitive recovery in APP/PS1 transgenic mice by co-administration of EPPS and donepezil. *Sci. Rep*. **6**, 34165; doi: 10.1038/srep34165 (2016).

## Supplementary Material

Supplementary Information
