# Supplementary material for: Rapid and sustained cognitive recovery in APP/PS1 transgenic mice by co-administration of EPPS and donepezil
Source: Sci Rep. 2016 Oct 31;6:34165. doi: 10.1038/srep34165 (PMC5086916; doi:10.1038/srep34165)
Supplement: Supplementary Information [file srep34165-s1.doc]

Rapid and sustained cognitive recovery in APP/PS1 transgenic mice by co-administration of EPPS and donepezil

Hye Yun Kim^1,2,3^,*, Hyunjin Vincent Kim^1,2^,*, Dongkeun K. Lee^1^, Seung-Hoon Yang^1^ & YoungSoo Kim^1,2^

^1^Convergence Research Center for Dementia and Center for Neuro-Medicine, Brain Science Institute, Korea Institute of Science and Technology, Hwarangno 14-gil 5, Seongbuk-gu, Seoul, Republic of Korea, ^2^Biological Chemistry Program, Korea University of Science and Technology, 217 Gajungro, Yuseong-gu, Daejeon, Republic of Korea, ^3^Research Institute, GoshenBiotech, Inc., 83-2 Wolmun-ri, Wabu-eup, Namyangju-si, Gyeonggi-do, Republic of Korea.

*These authors contributed equally to this work

Correspondence and requests for materials should be addressed to Y.K. (yskim@bio.kist.re.kr)

**Supplementary Table 1. Statistical analyses of weekly Y-maze tests during EPPS administrations.**

**Supplementary Table 2. Statistical analyses of weekly Y-maze tests during donepezil administrations.**

**Supplementary Table 3. Statistical analyses of weekly Y-maze tests during EPPS/donepezil co-administrations.**
